# Practical Observations on Nervous and Sympathetic Palpitation of the Heart, Particularly as Distinguished from Palpitation, the Result of Organic Disease, &c. &c.

**Published:** 1838-04-01

**Authors:** 


### Practical Observations on Nervous and Sympathetic Palpitation of the Heart, particularly as distinguished from Palpitation the Result of Organic Disease; to which are prefixed some General Remarks on the Use of the Stethoscope, and Employment of Percussion in Diagnosis of Diseases of the Heart and Lungs. By *John Calthrop Williams,* M.D. Physician to the Nottingham Dispensary, &c. &c. London, 1836.

Among the arguments adduced in favor of Provincial Medical Schools, we do not remember having seen any notice taken of their beneficial effects as regards the teachers themselves. Urgently called upon to make themselves masters of the subjects which they are to discuss, they are led to study, and think upon them much more intently than they would otherwise do. In this way the number of labourers in the field of science is increased, and men who might, but for the application of such a stimulus, have plodded thoughtlessly on along the beaten road of practice, become careful explorers and observers, and consequently safe guides for the conducting of others.

These remarks are elicited by the fact that the volume before us consists, as is stated in the Preface, of observations which originally formed the substance of part of a course of lectures on the principles and practice of physic, delivered, about three years ago, in the then newly-established medical school of Nottingham.

The work consists, as its title indicates, of two parts. The former, occupying a space of forty-three pages, treats of the general use of the stethoscope; and might, we think, have been altogether omitted, as it contains nothing new, and is, in fact, little else than a compilation from other authors. We cannot help also remarking that the figure of the stethoscope, delineated at the beginning of the work, looks (at the present day, when the instrument is so generally and so well known) too much like vain parade. An ill-natured person might, with some show of reason, insist that it was meant for *a trumpet*, to which instrument indeed it bears no inconsiderable resemblance. But let not these remarks prejudice us in our consideration and estimate of that other part of the work, to which we mean to draw the attention of our readers.

The part here alluded to, may be divided into two sections; the first of which treats of Sympathy in general, the other of "Nervous and Sympathetic Palpitation of the Heart.

After stating numerous familiar examples of sympathy, our author says:—

"Sympathies may be divided into *general* or *universal*—*particular* or *specific*. They may likewise be regarded as *natural* or *morbid*—or sympathies may be divided into such as manifest themselves on the organs of *volition*, such as result from *instinctive propensities* or *instinct;* and those which proceed from *emotions* and *sensations*.

Those sympathies which belong to the two former heads of this division, operate through the medium of the motor nerves, if we except some few examples referable to instinct solely, which manifest themselves on the muscles of voluntary motion. They may be said to form the chain of communication between the brain and the corporeal organs, and thus to enable these latter to obey the dictates of the will, and administer to the necessities of life.

Sympathies resulting from *emotions* and *sensations*, on the other hand, declare themselves through the medium of a much more equivocal agent. They manifest themselves not only on the organs of volition, but also on those of *involuntary* motion, as well as on others destined to perform the functions of *secretion*. The effects they produce on the organs of volition are intimately allied to such as arise from instinctive propensities, inasmuch as they are often produced independent of the will. *Morbid* sympathies may operate in all the foregoing manners, but are principally associated with our sensations and emotions. They often are the result of local or general irritations, applied from without, or generated within the body, and in some instances are calculated to affect the functions only; in others they affect the vital actions of the parts in which they happen to be seated.

To the class of sympathies that manifest themselves through the intervention of the organs of volition and instinct, belong all the decided effects produced on the voluntary agents of the body, which emanate from *consciousness*. There is this difference, however, between the purely voluntary, and instinctive sympathies: the first result from the circuitous process of imagination, reasoning, and association, and may be easily traced; while the latter, proceeding *not* from consciousness, but from the sensations excited by animal appetites, and innate propensities, cannot be always mentally traced. Their physical effects, moreover, are *instantaneous*, *inevitable* and *perfect* from the first; while the former require time and practice to bring them to perfection. In other words, the instinctive sympathies operate independent of reason, even prior to its dawn; the voluntary associations are dependent upon this power.

The sympathies of *emotion* seem to influence primarily the whole nervous system, and subsequently the heart and circulation, and the secretory organs and other parts, which perform their functions independent of the will. This operation they apparently effect through the intervention of that communication which exists between the brain and the *great intercostal nerves*, in the course of the cerebro-spinal axis. Thus is explained the simultaneous events that proceed from anger, hope, fear, grief, joy, and the like, which are apparent at one and the same instant in the nervous and circulatory systems.

The sympathies arising from *sensation*, likewise, operate in many instances, on the sanguineous system, more especially those referable to the *morbid* class; while in others, they affect the muscles, and produce involuntary motions of the voluntary organs. Thus pain creates an increased action of the heart and arteries, and is soon accompanied by all the phenomena of fever: and the sensation produced by tickling the soles of the feet, induces spasmodic excitation of the muscles of respiration, and *laughter* is the result; which, if carried to excess, in an irritable temperament, might be succeeded by convulsions of a more serious nature.—These, and innumerable other examples, are common-place matter of fact, with which most persons are familiar; but with 'regard to the vinculum,' as Dr. Wilson aptly observes, in his philosophical and learned letters on the action of morbid sympathies, 'that connects the sympathising part with that where the irritation is applied, we remain totally in the dark.'—What we have in our power, then, is by careful observation to mark truly demonstrable facts, and the knowledge of these will lead us to more correct practice." 49.

Dr. Williams very properly deprecates the too prevalent rage for tracing diseases almost exclusively to *vascular* derangement. "No man," he remarks, "can be a sound pathologist, or a judicious practitioner, who devotes his attention to one of these systems (the vascular and nervous) in preference or to the exclusion of the other; through life they are perpetually acting, and inseparably linked together. The laws of *morbid* sympathy, moreover, are often manifest to the senses; we have, therefore, every ground to infer, that they may be reasoned upon in diagnostics with the utmost propriety, and considered as steady principles for the guidance of our practice.

Darwin, the great father of the sympathetic doctrines of modern times, was said to be the most successful practitioner that England ever produced. There was scarcely a town on the Continent from whence he was not consulted, either personally or by letter; and we have it from good authority, that his success was mainly owing to the multiplied resources at his command, from the sympathetic views of disease he maintained."

With reference to the latter paragraph of this quotation, we beg to state our conviction (and we know something of Darwin's practice from having frequently conversed on the subject with more than one intelligent patient of his) that his success was attributable mainly—not as our author thinks to the "multiplied resources at his command, from the sympathetic views of disease maintained"—but to his good sense in never letting his fanciful, though ingenious theoretical views influence, in any considerable degree, his practice.

Adverting to the sympathetic influence exercised by the stomach over the heart, our author says:

"My own experience supports the opinion of those who maintain that the symptoms of Angina Pectoris may be present, and frequently are present, without organic disease of the heart, but dependent upon morbid sympathy, arising from functional derangement of other organs, more especially the stomach.

The following case of the late Dr. Marsden, of this town, though not precisely proving the above position, is still an interesting one; for though the heart was diseased, I think the paroxysms were excited by sympathy, and in a great measure the consequence of occasional disorder of the stomach. He was irregular in his diet, which materially interrupted the healthy action of the digestive organs, and ultimately aggravated the sympathetic paroxysms."

"About twelve years ago, Dr. Marsden, then aged 56, and in good health, ate at a friend's house, some suet dumpling—the suet of which was rancid, and disordered his stomach.

He suffered violent pain at the epigastrium. He pursued an abstemious diet, chiefly of fish, for many weeks, and gently affected the system with Mercury; during all which time he was engaged in extensive practice, and frequently exposed to cold. He was no better: the pain recurring at irregular intervals.

About two years after the first attack, he was riding a spirited horse, which started at some object; and the agitation of this accident suddenly brought on the pain in the epigastrium, which then, for the first time, mounted towards the throat, and descended along each arm. Ever since then, he was unable to mount a hill, or stair, without difficulty and pain; nor could he endure pressure on either arm—as when walking in company.

Gradually the violence of the pain diminished at the epigastrium, and ascended to the larynx, along the course of the recurrent nerves. He could not stoop, nor effect any evacuation, nor undergo any bodily exertion nor mental emotion, without a more or less severe attack of pain.

Thus the paroxysms continued to the time of his death; they *did not* impede respiration more than any other bodily pain:—they utterly prostrated his strength during the attack, and left much languor on their subsidence. There latterly appeared a tendency to recur about midnight; but any indiscretion in diet, of quality or quantity, was sure to accelerate, protract, and aggravate the attack.

He was seldom able to lie on his left side, without the recurrence of the pain: his bowels were costive, his flesh attenuated, his complexion bilious. His appetite, thirst, tongue, urine were natural. His pulse, about 60, small, easily compressible, intermittent, unless when above 70, then regular. The intermission commenced after a dose of Elaterium, nine years ago. He never had any palpitation until a few days before death.

He was stethoscoped by Dr. Sims, seven years ago, but nothing abnormal could be detected in the heart, aorta, or lungs.

About five months before his death, I, on two occasions, observed a sound between bruit de soufflet and rape, apparently in the mitral valves. The epigastrium presented a palpable fulness and hardness, giving the idea of pressing a piece of leather; the edge of the liver was distinctly palpable and very firm below the edge of the ribs. There was a slight fulness over the cæcum. Occasionally an odd sensation came on, which Dr. M. called the fidgets in his feet—apparently a tingling sensation—which rendered him extremely restless; was allayed by no application, and had no connexion with any observable cause.

Dr. Marsden never had rheumatism nor gout; but his father and brother were gouty.

Autopsy. The whole body was greatly emaciated: in the cavity of the left pleura were twelve ounces of bloody serum. In right ditto, sixteen ounces. There was no adhesion of the lungs; some emphysema at the edges, and considerable œdema of their substances. There was a cicatrix at the apex of each lung.

There was six or eight drachms of bloody serum in the cavity of the pericardium. Heart very flabby; its substance very easily lacerable. An adhesion of elongated fibres, one inch and a quarter long, and half an inch broad, along the junction of the ventricles, and a smaller one of the same kind, but with longer fibres, at the apex of the left ventricle—both of old-standing. A thin adhesion of the left auricle and pericardium. The left ventricle was dilated to twice its natural size, with walls of ordinary thickness. Left auricle of natural size and structure; mitral valves somewhat thickened—chordæ tendineæ decidedly hypertrophied. The heart a little hypertrophied (passively), in all its other dimensions. The aortic valves thickened at the base; the sesamoid bodies hardened and enlarged, and covered with several very small cauliflower and linear excrescences. The coronary arteries pervious, perfectly ossified as far as their subdivision. The ossific formation scratches the nail. Patches of artheromatous matter in the ascending aorta, and one large hard ossification upon the smaller and lower circumference of its arch, embracing about a third of the circumference of the vessel. A patch of artheroma at the origin of the mesenteric artery: a ring of ossification surrounding the origin of the splenic artery, which is given off from the aorta above the cæliac axis; an extended artheromatous layer around the cæliac artery. From this point, the patches of ossification on the aorta increased to its bifurcation and were traced as far as the right external and internal iliacs. The internal membrane of the artery from the cæliac to the iliacs, was absorbed over the *edges* of the ossific patches, and, where not absorbed, was of a slate colour. The spleen was small and firm—its peritoneal coat thickened. The liver of natural size, firm, considerably congested, and the lobulus spigelii, particularly so. The pancreas abnormally soft. The right kidney marbled with several patches of disease, exhibiting deficient vascularity. On the surface of the left kidney, some urinary vesiculæ. In both kidneys, traces of deposition of albuminous matter in the tubular portion.

The stomach was distinctly divided into a cardiac and pyloric portion, by a transverse constriction; its capacity considerable, the mucous membrane firm and undigested, and very much conjested about the cardiac portion. The whole stomach was thick and firm, and on the lesser curvature was an old cicatrix. The mucous membrane of the duodenum was congested and firm; the mucous membrane of the cæcum excessively congested, so as to be quite *black*—the mucous membrane of the ileum presented much the same appearance; no other pathological appearances were observed. The head was not examined." 58.

The foregoing case, which is, in many respects, an interesting one, was drawn up by Mr. Greeves, a friend of Dr. Williams, and is regarded by the latter as "a compound case of organic disease and nervous sympathy: one wherein I should think it probable, that the disordered functions of the stomach first induced deranged action of the heart, and that this in turn degenerated into absolute organic disease, which was ever after under the dominion of the first cause. It is illustrative of the great importance of paying strict attention to diet, and of preserving in healthy action the digestive organs."

The extensive influence which a disordered state of the digestive apparatus in general, and of the stomach in particular, exercises over the whole frame, mental and corporeal, has long been, and will still continue to be, a subject of wonder and admiration to pathologists. This influence is undoubtedly reciprocal, but the sympathies which radiate *from* the gastric centre, are more powerful than those which are propagated *to it* from remote organs. The chief if not the only medium of these sympathies is the *intercostal or ganglionic system of nerves*, a system in close connexion with, but not solely dependent on the brain, co-operating but having a separate existence. Its centre is the semi-lunar ganglion and solar plexus, which are in close proximity with the stomach, and liberally supply it with nerves; and hence, in all probability, the reason why a severe blow over the stomach proves so often suddenly fatal.

"The *par vagum, phrenic, glosso-pharyngeal,* and *spinal accessary* nerves, appear to be all appropriated solely to supply the power of performing the respective functions of the individual parts on which they are ramified ; and their frequent junction with the sympathetics, is only an evidence of the great necessity there is of preserving a perfect accordance among the organs, structures, and viscera essential to animal life." 62.

After these general remarks on sympathy, which in the work before us occupy the space of 21 pages, we come to that section which treats of "Nervous and Sympathetic Palpitation of the Heart," a subject certainly of considerable interest. The following passage from the posthumous writings of Dr. Baillie, and which is quoted by our author in his "Preliminary Observations," contains such an important, and often necessary caution, that we cannot refrain from transcribing it.

"There are in truth few phenomena, which puzzle, perplex, and lead into error the inexperienced (and sometimes the experienced) practitioner, so much as inordinate action of the heart. He sees, or thinks he sees, some terrible cause for this tumult in the central organ of the circulation, and frames his portentous diagnosis and prognosis accordingly. In the pride of his penetration he renders miserable for a time the friends—and by his direful countenance damps the spirits of his patient. But ultimate recovery not seldom *disappoints* his fears, and the Physician is *mortified* at his own success."

Palpitation is defined by our author as a temporary augmentation of the frequency in the action of the heart (with or without irregularity), the pulsations of the organ being at the same time perceptible to the individual. *Nervous* palpitation presents itself in two distinct forms, *active* and *passive.* The first occurs when the system is in a state of *plethora ;* the second, when in a state of *exhaustion.* Of the plethoric form the following instance is given.

"A stout, ruddy-faced, plethoric country girl consulted me not very long since, in consequence of the extreme inconvenience she experienced from the over-action of her heart. It beat full and strong against her ribs, and with sufficient force sensibly to raise my hand at each stroke, when the stethoscope was applied over the præcordial region, though this is not usually the case. To this constant state of inordinate action were added occasional attacks of palpitation, so tumultuous as scarcely to admit of analysis. On enquiry, I found the uterus had not performed its accustomed healthy function for several periods ; and in consequence, she had become feverish and irritable; the heart, in turn, had sympathised with the general derangement of the system, but she complained only of the palpitation.

I was quite satisfied of the sympathetic nature of the affection, and by ordering great quietude, low diet, blood-letting from the arm in the first instance, and then over the loins and above the pubes, by means of cupping and the application of leeches, with the exhibition of active purgatives, I soon restored her to health."

The case above related, might at first sight be mistaken for one of hypertrophy of heart; but in this disease, the impulse of the heart is hard, circumscribed, and heavy ; it raises the head of the observer at each stroke. The sound, instead of being clear, is obscure, and as it were muffled. A careful examination with the stethoscope will generally detect some irregularity in the contractions of the various chambers of the heart, either in respect to order or force. Percussion over the præcordial region, in advanced cases produces a dull sound, which is not met with when the dimensions of the organ are natural. In hypertrophy, when the palpitations are, pro tempore, suspended, the beat of the heart still remains hard and more circumscribed than it ought to be, and indeed the movements of the heart are not always of a nature to merit the name of palpitation; they are not necessarily more rapid than in health—the patient does not always perceive them himself—and when he does, although they may in reality be much more forcible than natural, he seldom complains of the sensation being a source of distress, unless when the capacity of the chest is diminished by pressure, position, or otherwise. He attaches no undue importance to his symptoms, he evinces no unnecessary alarm respecting them, and even in the advanced stages of the complaint, seems insensible to his danger.

Passive palpitation of the heart is more frequent and obstinate than the active or plethoric form. It is produced by a transient suspension of the contractions of the heart, followed by a forced effort to recover its equilibrium of action. The palpitations, though sensibly felt by the patient, are scarcely perceptible on examination. The impulse of the heart is not increased; sometimes it is even weaker than natural, a fact noticed by Bertin. Though this form of palpitation is attended with no immediate danger, yet we are assured by Foderé, that those who have been so affected from youth upwards, commonly die before the usual term of human existence. In slight cases of passive palpitation, the action of the heart is compared by the patient to the flutter of a bird; sometimes it consists of a momentary feeling of a rolling or tumbling motion of the heart, accompanied by an intermission of the pulse, of which the patient himself is often conscious. This perceptibility on the part of the patient is thought by Dr. Joseph Brown to be *pathognomic* of nervous or sympathetic intermission of the pulse, and has been noticed by Dr. Jas. Johnson, and other authors. The pulse is irregular when the action of the heart itself is irregular; but frequently there is no irregularity of action, the affection consisting merely of a pulsation more or less strong, which the patient feels, or hears perhaps, and can count distinctly, especially when lying in bed. The palpitations recur after irregular intervals, and are uncertain in their period of continuance. The breathing during a paroxysm is slightly hurried, with a sense of anxiety, and a feeling of apprehension, on the part of the patient, greater than the real danger justifies. The sounds of the heart are soft and clear, and loud when compared with the impulse. The pulse is sharp and jerking, never full and strong. There are besides neither symptoms of determination to the head, nor of impeded general circulation, such as lividity of countenance, dropsical effusions, &c. The absence of these, and certain other symptoms, sufficiently distinguishes the affection from organic disease of the heart. The diagnosis may be assisted by taking into consideration the temperament, as well as the age of the patient. In hysterical and hypochondriacal patients, nervous palpitations are very common: they occur also frequently in the young and sanguine, between the age of 15 and 25 years. Organic disease, on the other hand, is rarely seen until the prime of life is past. As a further ground of distinction Dr. Williams asserts, that while anti-spasmodics and stimulants afford relief in nervous palpitation, they greatly aggravate the patient's sufferings when there is organic disease. This statement is anything but correct. We have scarcely ever met with an instance of organic disease of the heart, in which the patient's sufferings were not temporarily relieved by stimulants. The propriety of administering them frequently in such disease, is altogether another question, and one on the considera-tion of which we cannot at present enter. We may state however our con-viction, that in many cases of organic disease they may be employed with positive and very marked advantage.

"Certain complications of disordered function usually connected with the palpitation which accompanies absolute disease of the heart, may, from acci-dental circumstances, co-exist with nervous irregularity of the actions of this organ. Of these may be mentioned the intermittent "bruit de soufflet," or bellows-sound, and the sawing murmur; both of which are to be attributed to the morbid velocity with which the blood is propelled by the sudden, and as it were, spasmodic contraction of the cavities of the heart. The murmur, accord-ing to Dr. Hope, occurs whenever the movements of the organ are accelerated, and in some instances the slightest causes produce that effect; as, a momentary emotion, a constrained position, or a change of posture from the recumbent position to the erect; a full meal, flatus on the stomach, &c. Some of these, by altering the position of the heart, may offer a trifling impediment to the free exit of blood, and thus perhaps contribute to produce the phenomenon in question."

Laennec states, that the bellows-sound and purring may often be detected in the course of the larger arteries, and Dr. Williams' own observation leads him to concur in the remark. Pricking pain, felt over the præcordial region, is another frequent sign of nervous derangement of function. Dr. Elliotson has noticed this symptom.

"Another peculiarity of nervous palpitation that occasionally presents itself is the perception of a double movement in the heart. When lying on his left side, the patient in such cases describes the pulsations of the organ as double those of the pulse felt in any of the arteries. This arises from his morbid state of irritability, rendering the contraction of the auricles also apparent to his senses. From Sir Benjamin Brodie's and Dr. Hope's experiments, there is reason to believe, that the contraction of the auricles can in no instance be strong enough to admit of detection, unless when the heart can be seen or touched. A certain degree of oppression and dyspnœa usually accompanies this variety of palpitation, and may vary from a mere feeling of anxiety, with accele-rated respiration, to perfect orthopnœa. This double movement is, however, sometimes dependent on a species of intermission, and in certain cases of dis-eased heart, is productive of curious results. Every second pulsation may be so feeble, as to be almost imperceptible. In the former case, the pulse appears to be quite regular and slow; but while in the act of feeling it, the intermediate or latent pulsation becomes suddenly distinct, and the pulse appears to be in-stantly doubled. A case of this description is recorded by Dr. Forbes, wherein the same patient had a regular pulse at fifty or sixty, and a regular one at one hundred, and one hundred and twenty, within the space of three minutes. Dr. James Johnson was, however, I believe, the first who really pointed out this species of intermission several years ago, in the case of a gentleman resid-ing in London, under the care of Mr. Cosgreave. In this instance, he informs us, 'that the ventricular actions were usually double those of the tangible ar-teries. But when any feverish excitement took place, the pulse became double the number, or more, at the wrist, and corresponded exactly with the pulsations of the heart against the ribs.'" 83.

With a view to illustrate more clearly the diagnosis of nervous palpi-tation, and thus to establish on a sure basis the principles of treatment, six cases of the affection are related, in addition to the one which we have already transcribed. The first two were cases of hysterical palpitation, and were both cured by means of aperient and tonic medicines, aided by judicious general treatment. The third case was that of a gentleman, not quite 40 years of age, who had been unsuccessful in business, and had become, in consequence, low and desponding. His digestive organs were much deranged, and he laboured under a continued, obscure masked fever. He complained occasionally of paroxysms of palpitation, which were accompanied with dyspnœa, insomnolency, and a distressing feeling of anxiety in the præcordial region. In this state he remained several months. He was then affected with hæmoptysis; and symptoms of subacute pericarditis supervened. On the cessation of the hæmoptysis, he was harassed with a troublesome cough, attended by copious expectoration of a somewhat purulent character. During the existence of these latter symptoms, he was several times affected with fits of syncope, which occurred without any evident cause. His father was reported to have died of diseased heart. He himself had been affected with epilepsy in his youth, and at thirteen years of age had suffered from St. Vitus's dance. He was at first purged with calomel and colocynth, properly dieted, and subjected to a course of anti-spasmodics and digitalis. On the supervention of the inflammatory symptoms, he was largely leeched and blistered. He continued also to take the digitalis, and was put upon a gentle mercurial course. In about a month the inflammatory symptoms yielded but the palpitation continued. He *again* (we find no previous notice of these remedies) had recourse to bitters and mineral tonics, and ultimately recovered.

On this case we are presented with the following observations.

"The history of the case declares its character to have been at first a nervous spasmodic palpitation, symptomatic of the enervated condition of the whole system, on which supervened subacute pericarditis; and the treatment was in accordance with this view of the disease. The condition of the primæ viæ, and his state of mental depression, although primarily induced by misfortune, yet in a great measure kept up and aggravated by the derangement of the alimentary canal, claimed first attention." 90.

"Palpitation," continues our author, "is found more frequently connected with dyspepsia, and derangement of the first passages generally, than with any other disorder. I have known nervous and excitable individuals experience the most severe paroxysms in consequence of having eaten too freely, either of general food, or of any article of diet, which, from pecularity of constitution, was difficult of digestion. In some of these cases, indeed, the distress will continue long after the offending cause is removed, and occasionally assumes a degree of obstinacy unaccountable and almost incredible. When dependent on chronic, or intractable disease of the primæ viæ, the severity of form it may assume is quite astonishing." 92.

We quite agree with Dr. Williams in attributing to a disordered state of the digestive organs, so much influence in the production of symptomatic palpitation of the heart. We feel assured that a judicious and *persevering* use of purgatives, in cases such as that just related, does more to restore health than any other plan of treatment whatsoever.

Among the sources of irritation to which the intestinal canal is exposed, there is none of which abnormal action of the heart is a more frequent and obstinate attendant than vermination, particularly when the parasite is a tape-worm. This remark is well illustrated by Dr. Williams's fifth case. The patient, a young lady, 22 years of age, was relieved from most distressing paroxysms of palpitation by oil of turpentine and other active purgatives, under the operation of which she voided, in the space of fourteen months, many yards of tape-worm.

"But of all the organs in the body, in whose functional irregularities the heart is prone to participate, the uterus stands, perhaps, the most prominent. Dysmenorrhœa, or total suppression of the menstrual discharge, is very frequently attended by palpitations. In the stout plethoric girl the affection is usually of the active kind, and may be attended with many other symptoms indicative of the full habit thus induced.—The following case will serve very well to illustrate the nature and ordinary phenomena of such an attack:—

CASE 6. During menstruation, a stout plethoric girl had some cold water spilled accidentally over her legs and feet. The secretion ceased in a few hours afterwards, and did not return for several periods. In the mean time she became affected with pain in the loins and thighs, headache and giddiness, and general febrile irritation. She was attacked with hysteric epilepsy; the heart sympathised; she had palpitation and other symptoms of constitutional disturbance. On listening to the action of the heart, it did not beat over a greater extent than natural, and there was no dull sound on striking over the præcordial region, more than usual. There was no preternatural sound accompanying the contraction of the cavities, and the increased action, constituting the palpitation, was universal. In this case there was, of course, no organic disease of the heart, and the administration of such remedies as relieved the congested system, generally and locally, and restored the menstrual discharge, dismissed the girl's palpitation, and induced health." 98.

In chlorotic females, the affection assumes the *passive* character; but the quick irritable pulse, &c. may mislead the routine practitioner, diverting his attention from the real and primitive seat of the disorder, the *uterine system*, and directing it to the affection of the heart, which is merely sympathetic. This is Dr. Williams's statement, and view of the case. Our own impression is, that the affection of the uterus is as much a symptom, as that of the heart itself. Chlorosis is a disease of the whole system, affecting, in a greater or less degree, every organ, and indeed the circulating fluid itself. It is high time that those partial notions of disease, which attributed such a potent and mysterious influence to the uterus, should give way to more comprehensive views, and sounder pathological principles.

On the cessation of the menses there is produced in some females troublesome and obstinate palpitation, which may be either of the active or the passive kind. This distinction must be duly attended to in instituting a plan of treatment, as the remedies which cure the one form, never fail to aggravate the other.

Dr. Williams alludes to the alleged influence of *spinal irritation* in producing paroxysms of palpitation; but it is not very clear what his own opinions on the subject are. "I have witnessed," says he, "cases of this kind, but not frequently; and I am satisfied it may materially influence existing disorder of the heart's functions, emanating from other sources. I am not prepared to allow, that the power which the ganglionic system of nerves possesses, primarily and wholly proceeds from the brain, though intimately connected with, and in some measure dependent upon the cerebral system; and as the nerves of *sensation* that run directly from the spinal chord to the central organ of circulation, are comparatively few in number, I apprehend, that when diseased, either at their origin, in their course, or ultimate ramifications, they are more likely to produce painful sensations than derangement of function; admitting, still, that ultimately both disordered function and diseased structure may be induced, for impaired energy of any part of the nervous system must materially influence an organ dependent upon it for health and energy. I have been enabled to trace palpitations to spinal irritation, but I think the phenomena were induced through the intervention of the cervical ganglionic nerves."

The first sentence of the foregoing quotation is very clumsily arranged; and the whole paragraph is a good specimen of what may be called, "writing on both sides of the question." Our own opinion on the subject is very decided. Of all the theories which a warm fancy, brooding over a number of one-eyed observations, has ever hatched and nurtured, we know not one, which, for the period of its existence, has been productive of so much mischief, as the fashionable doctrine of "spinal irritation." Give us one of the healthiest and most ruddy-faced of country girls, and we will undertake, in a very few weeks, to make her back exquisitely sensitive, and her general nervous system in the highest degree irritable, by leeching and cupping her in strict compliance with the most approved principles laid down by the advocates of "spinal irritation."

Rheumatic pericarditis is briefly, and very imperfectly alluded to as a cause of palpitation. No notice is taken of one of its consequences, which, when it exists, is a permanent cause of disordered action of the heart—we mean adhesion of the pericardium.

Palpitation is often produced by manustupration. Dr. Williams details the accompanying symptoms occasioned by this most baneful habit, but we do not feel ourselves called upon to do more than merely allude to the subject. The following remarks however, which are of more general application, we deem it our duty to transcribe, inasmuch as while treating of the diagnosis of *functional* palpitation, we did not, perhaps, dwell sufficiently on the physical signs.

"When derangement of the heart's action is strictly functional in its nature, and dependent on the sympathetic consent between it and disease seated primarily in some distant part, our diagnosis will be materially aided by attending to the following particulars:—We must ascertain by the hand, ear, and Stethoscope, whether the abnormal action of the organ be universal or not, over the whole extent of the præcordial region. If it be found on both sides of the heart, and not on one part more than another, it leads to the conclusion that the palpitation is merely a functional disturbance; but this does not constitute positive proof. We must examine, at the same time, by striking over the region of the heart, either by the fingers, or through the medium of the Pleximeter, whether the dull sound peculiar to this region be not of greater extent than natural. This induces the conclusion that there is no dilatation, nor effusion into the pericardium.

It was correctly remarked by Dr. Elliotson, in his Lectures: 'If at the same time that we observe the action to be violent, and yet the sound on Percussion not to be more extensive than usual; and in addition to this circumstance, we find the sound of the heart perfectly normal—no bellows-sound, no rasping, no sawing sound, merely perhaps a rather louder sound than usual—nothing more than that—then we have every reason to conclude, that there is no organic disease of the heart.'" 110.

The work concludes with a chapter on the treatment of palpitation; but it is little else than a *resumé* of the curative remarks scattered throughout the body of the work, and of which we have already noticed, in general terms, all the most important. We shall here therefore only add, that in the *plethoric* or *plethoro-nervous* form of palpitation, our author very properly recommends an efficient, though cautious abstraction of blood, both from the general system, and from the præcordial region. When the plethora is dependent upon suppression of the catamenial discharge, cupping-glasses applied to the sacrum, and leeches to the inside of the thighs or feet, are likely to prove beneficial.

We have already, at the commencement of our critique, stated our opinion of the design and plan of the work before us. We shall now make a few general observations on the manner of its execution.

Its most obvious and important defect is the want of a judicious and clear arrangement. The functional and organic diseases of the heart, their symptoms and treatment, the physical and general signs, are all jumbled together in strange confusion; while topics, half-discussed, are abandoned for others, and then resumed, in such a way as necessarily to produce frequent repetition. Thus the style is rendered loose and rambling, and the mind of the reader (unless he is previously well acquainted with the subjects treated) can scarcely form any definite notions of the principles, whether of diagnosis or treatment, which it is the author's main purpose to inculcate. We think too that many of the quotations might have been omitted, being either of little importance, or extracted from books which are in every body's hands. But with all these grounds for animadversion, the work contains many excellent practical precepts; and it is impossible to read it carefully, and not be convinced that Dr. Williams is a well-informed and discriminating physician.

